# High Resolution Switching Mode Inductance-to-Frequency Converter with Temperature Compensationti

**DOI:** 10.3390/s141019242

**Published:** 2014-10-16

**Authors:** Vojko Matko, Miro Milanović

**Affiliations:** 1 Institute for Automation, Faculty of Electrical Engineering and Computer Science, University of Maribor, Smetanova 17, 2000 Maribor, Slovenia; 2 Institute for Robotics, Faculty of Electrical Engineering and Computer Science, University of Maribor, Smetanova 17, 2000 Maribor, Slovenia; E-Mail: milanovic@um.si

**Keywords:** inductance-to-frequency converter with picoHenry resolution, switching method, dynamic temperature compensation of circuit elements, precision metrology

## Abstract

This article proposes a novel method for the temperature-compensated inductance-to-frequency converter with a single quartz crystal oscillating in the switching oscillating circuit to achieve better temperature stability of the converter. The novelty of this method lies in the switching-mode converter, the use of additionally connected impedances in parallel to the shunt capacitances of the quartz crystal, and two inductances in series to the quartz crystal. This brings a considerable reduction of the temperature influence of AT-cut crystal frequency change in the temperature range between 10 and 40 °C. The oscillator switching method and the switching impedances connected to the quartz crystal do not only compensate for the crystal's natural temperature characteristics but also any other influences on the crystal such as ageing as well as from other oscillating circuit elements. In addition, the method also improves frequency sensitivity in inductance measurements. The experimental results show that through high temperature compensation improvement of the quartz crystal characteristics, this switching method theoretically enables a 2 pH resolution. It converts inductance to frequency in the range of 85–100 μH to 2–560 kHz.

## 1. Introduction

Inductance-to-frequency conversion has become in recent years increasingly popular in a large variety of applications that are designed, for instance, for the measurement of a number of physical measurands, such as mechanical displacement, nanopositioning, eccentric motion, and strain sensing [1–3], liquid levels, pressure, *etc*. Inductance-to-frequency conversion is also used in biosensors in medical and electromagnetic material properties measurements. Typically, in many of them, inductance is first converted to the frequency signal and after that to physical or chemical quantity for analysis. High-resolution inductance-to-frequency conversion is also a well-established technique in microscale converters for material properties sensing and represents a universal transduction mechanism for the measurements in which the inductance changes need to be measured with great precision.

Many research studies in recent years have focused, in particular, on the methods that would make precise inductance measurements in the range well below some μH possible. Inductive resolution plays a vital role in the nH range. The latter can be achieved, for instance, by means of four-port adjustable inductor bridge with 0.18 μm Complementary Metal-Oxide-Semiconductor (CMOS) technology on plastic. When operating near 3, 4, 7.5 and 9 GHz, it has a Q-factor of 6.5, 6.7, 8 and 11.5 and an inductance of 2.1, 1.6, 1.1, 0.6 nH [4]. Other methods to detect small inductance changes include: (i) simultaneous detection of the inductance and Q-factor value changes of the unusual flat coil-based MHz-range resonator, leading to the resonant frequency and amplitude changes of the oscillator [5]; (ii) the improved “LC resonator” method for high resolution measurements of magnetic-field penetration depth which achieves the improvement by replacing a solenoid testing coil by an open-flat coil driven by a tunnel diode oscillator of a low power and a highly stable frequency [6]; (iii) a dual-modulus of 2/3 injection-locked frequency divider with wide locking ranges using an active inductor as the resonance loop, and a tunable active-resistance, which has locking ranges from 1.5 to 2.05 GHz [7]; (iv) a measurement system based on the Digital Lock-In (DLI) technique using a non-inductive reference resistor with high thermal stability connected with the magnet in series where the voltages across the reference resistor and the magnet are used as measurement signals which are sampled synchronously by the Analog-to-Digital Converter and processed with a DLI amplifier algorithm [8]; (v) a low-temperature tunnel diode oscillator circuit whose performance allows measurement of changes in the resonant frequency of an LC circuit with a precision of 0.001 ppm detecting extremely small changes in a number of material properties such as thermal expansion, surface impedance, and electric and magnetic susceptibilities [9]; and (vi) a Complementary Metal-Oxide-Semiconductor (CMOS)-based Magnetoencephalography (MEG) acquisition system consisting of a small-sized high inductance coil sensor and an instrumentation amplifier [10,11]. Only some of the above methods made any significant analysis with regard to the dynamic temperature influence, ageing of the elements, and any other influences on the measurement error.

The new idea for inductance-to-frequency converter uses the switching oscillator circuit oscillating at 4 MHz with as high temperature stability and measurement resolution as possible. Such use improves both the frequency sensitivity and the linearity of the characteristics. It also compensates the quartz crystal self-temperature-frequency characteristic, enabling a very stable converter functioning in an extended temperature range. In addition, it also strongly reduces the influence of the supply voltage on the oscillating circuit output signal, and foresees the functioning of the sensitive inductance element (in case where impedances are inductances) [12–15]. With regard to the temperature, the new method makes possible a stable functioning of the small inductance conversion to a frequency signal with a small number of elements in a converter (without any additional lock-in amplifier or host system and temperature sensor). In comparison to the above-mentioned methods [4–11], it is also cheaper and more suitable for industrial use.

Moreover, when additionally compared to some other methods [16–28] for the conversion of inductance to frequency, the newly proposed method also proved to have high dynamic stability during the temperature changes in the extended operating range when the temperature varies between 10 and 40 °C. The use of switching circuits in many instances improves electrical circuit characteristics (possibility to use quartz crystals with different cutting angles) and/or compensates (strongly reduces) certain influences [29–38].

## 2. Inductance-to-Frequency Converter Circuit

The proposed switching mode converter is based on an oscillator circuit and the symmetrical switching part (for the reduction of the temperature influence), as well as an additional circuit for the conversion of a 4 MHz signal to a lower kHz frequency range. The method does not require stable temperature. It operates well at changing temperatures, which allows access to crystal's pins and consequently the changing of the electrical quartz crystal substitution model. It is noteworthy to mention that an oscillator with a good start-up, *i.e.*, with a reliable crystal oscillation during the start and later on, is a must [39–44].

### 2.1. Converter Principle

**Figure 1.**
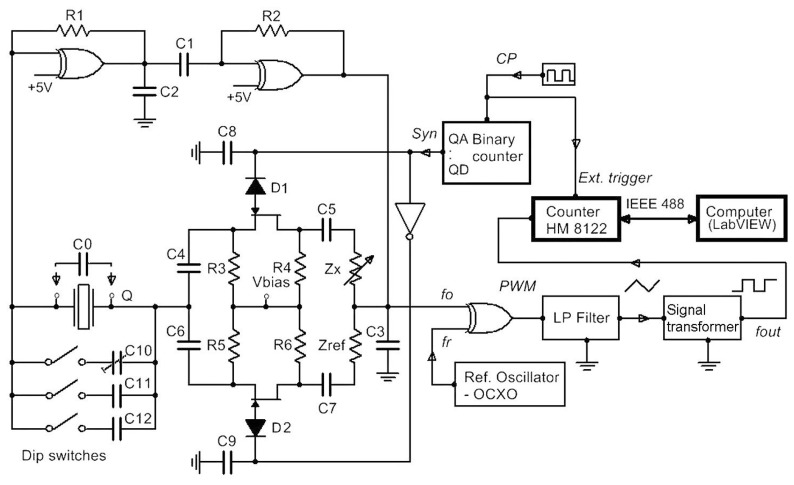
Schematic representation of the inductance-to-frequency converter.

The novelty of the method described in this article lies in the use of specific symmetrical switching mode oscillator and additionally connected impedances *Z*_x_ and *Z*_ref_ in series with Junction Gate Field-Effect Transistors (JFET) to the quartz crystal which has shunt capacitance *C*_0_ (Figure 1). Additional shunt capacitances *C*_10_–*C*_12_ are connected to the quartz crystal in parallel for the experimental stepwise increase of the capacitance *C*_0_. The conversion impedance *Z*_x_ and reference impedance *Z*_ref_ are connected to the quartz crystal in series and enable a significant reduction of the temperature influence on the frequency change because of symmetry of the circuit. This yields high frequency sensitivity and simultaneous compensation (high reduction) of all other disturbing influences. The switching between the frequencies *f*_01_ and *f*_02_ is performed through the switching signal (*Syn,* which can be 1 or 0) and an additional inverter circuit. The signal corresponding to the frequency difference between the frequency *f*_01_ and reference frequency *f*_r_ or difference between the frequency *f*_02_ and reference frequency *f*_r_ enters the LP filter (which is a pulse wide modulated signal) [45–47]. With the help of the reference frequency *f*_r_, both frequencies *f*_01_ and *f*_02_ (≅4 MHz) are converted to the range between 2 and 100 kHz, which is suitable for further signal processing. At the LP filter (with the response time of 4 μs) output, the triangular signal (with the initial setting frequency of 2 kHz depending on *C*_10_ and *Z*_ref_) is produced and then converted to a rectangular signal by a signal transforming circuit representing the output signal. The output *f*_out_ thus represents the temperature and any other influence compensated frequency signal which is synchronously measured with regard to the switching frequency which can change in the range *f*_Syn_ = 1–50 Hz (thus the converter response time is 0.02–1 s). Capacitances *C*_2_ and *C*_3_ serve to suppress the spurious responses to avoid crystal oscillation at higher or lower frequencies [12].

### 2.2. Expanded Use of the Inductance-to-Frequency Converter

The quartz stray capacitance *C*_0_ includes pin-to-pin input and output capacitance of the oscillator at the crystal pins, plus any parasitic capacitances. The typical value of the stray capacitance is between 1.5 pF and 5 pF. The connection of an additional stray capacitance connected in parallel to the quartz crystal, and of inductance *L*_x_ in series with the quartz crystal expands the possibility of the use of a frequency stable quartz crystal oscillator by influencing quartz crystal equivalent circuit as an inductive converter whose inductance is in the range of 85–100 μH. Stable oscillation, good linearity and high sensitivity in this range [48–52] are thus one of this method's major advantages. The crystal used in the experiment (Figure 1) was AT-cut [16] crystal with the temperature change ±5 ppm in the range of 0–50 °C. The data of the electrical quartz crystal equivalent elements are *f*_0_ = 4 MHz, *R* = 10 Ohm, *C* = 25 fF, *L* = 64 mH, *C*_o_ = 4 pF, quality *Q* = 80 k. The frequency *f*_0_ was selected due to a greater oscillation amplitude (at low frequency (under 10 MHz)) and a higher *Q* value for the selected oscillation circuit. The values in the quartz crystal equivalent circuit used in the experimental converter were measured by the HP4194A impedance/gain-phase analyzer. The JFET (N-channel) transistor (low level chopper) data are as follows: zero-bias G-D junction capacitance *C*_GD_ = 6.9 pF, zero-bias G-S junction capacitance *C*_GS_ = 9 pF, drain ohmic resistance *R*_D_ = 1 Ω, source ohmic resistance = 1 Ω, rise time = 2 ns, and fall time = 15 ns. Capacitances *C*_4_, *C*_5_, *C*_6_ and *C*_7_ are the same and equal 33 nF, *C*_8_ and *C*_9_ equal 1 nF. Resistors *R*_3_, *R*_4_, *R*_5_ and *R*_6_ are 1 MΩ (symmetrical elements in the circuit must be of the same material and of the same quality to assure the same temperature properties).

### 2.3. Temperature Compensation Using Switching Method

When impedances *Z*_x_ and *Z*_ref_ (Figure 1) are the same, *f*_01_ and *f*_02_ remain almost the same at states 1 and 0 of the *Syn* signal and depend on the quartz crystal resonant frequency *f*_0_, quartz crystal temperature characteristics *Δf*_0_ (*T*), its ageing *Δf*_0_ (*t*) and the *Z*_x_ and *Z*_ref_ inequality, as well as *Δf*_0_ (*ΔC*_0_eff_) change. However, when the impedances *Z*_x_ and *Z*_ref_ are different, the frequencies *f*_01_ and *f*_02_ depend on the state of *Syn*, the quartz crystal series resonant frequency *f*_0_, quartz crystal temperature characteristics *Δf*_0_ (*T*), its ageing *Δf*_0_ (*t*), impedances *Δf*_0_ (*Z*_x_) and *Δf*_0_ (*Z*_ref_), as well as *Δf*_0_ (*ΔC*_0_eff_) change In case of the difference of the two frequencies *f*_01_ and *f*_02,_ *Δf*_0_ (*T*)*, Δf*_0_ (*t*), and *Δf*_0_ (*ΔC*_0_eff_) are strongly reduced because only one temperature quartz characteristics is involved.

The output frequency *f*_out_ depends on *Syn* signal, *f*_0_ and reference frequency *f*_r_ and can be expanded to (for *Syn* = 1 and for *Syn* = 0 in case *Z*_x_ = *R*_x_ + j*ωL*_x_ and *Z*_ref_ = *R*_ref_ + j*ωL*_ref_, whereby when dealing with small inductance values, resistances *R*_x_ and *R*_ref_ can be ignored):

(1) $$f(Syn)-f_{r}=f_{0}+\Delta f_{0}(T_{1})+\Delta f_{0}(t_{1})+\Delta f_{0}(C_{0\_eff})+\Delta f_{0}(L_{x})-(f_{r1}(T_{1})+\Delta f_{r1}(T_{1}))+\Delta f_{c1}(cou\_err_{1})$$

(2) $$f(\bar{Syn})-f_{r}=f_{0}+\Delta f_{0}(T_{2})+\Delta f_{0}(t_{2})+\Delta f_{0}(C_{0\_eff})+\Delta f_{0}(L_{ref})-(f_{r2}(T_{2})+\Delta f_{r2}(T_{2}))+\Delta f_{c2}(cou\_err_{2})$$

where *Δf*_r_ (T) in Equations (1) and (2) represents the temperature instability of the reference oscillator signal and *Δf*_c_ (cou_err) a counter error [53]. The joining of *f*_0_ and *Δf*_0_ (*L*_x_) gives Equation (3) which represents *f*_01_. The particularity of this equation lies in the fact that it takes into account the compensations *C*_0_eff_ (Figure 1) and at the same time linearizes the quartz characteristics due to the *ΔL*_x_ change while allowing for the sensitivity setting (*k*) [15,30]

(3) $$f(Syn,L_{x})=\frac{1+\frac{C}{2\left(\frac{1}{k}C_{0\_eff}-\frac{1}{{\omega_{0}}^{2}\cdot k\cdot L_{x}}\right)}}{2\pi \cdot \sqrt{L\cdot C}}+\Delta f_{0}(T_{1})+\Delta f_{0}(t_{1})$$

where:

*L* and *C*—mechanical behavior of the crystal element,

*L*_x_—conversion inductance,

*C*_0_eff_ —sum of the actual parallel parasitic capacitances,

*k*—sensitivity value,

*f*_0_—quartz crystal series resonant frequency,

*T*—temperature,

*t*—time,

and *ω*_0_ is defined as Equation (4)

(4) $$\omega_{0}=2\pi f_{0}$$

The joining of *f*_0_ and *Δf*_0_ (*L*_ref_) gives Equation (5) which represents *f*_02_.

(5) $$f(\bar{Syn},L_{ref})=\frac{1+\frac{C}{2(\frac{1}{k}C_{0\_eff}-\frac{1}{{\omega_{0}}^{2}\cdot k\cdot L_{ref}})}}{2\pi \cdot \sqrt{L\cdot C}}+\Delta f_{0}(T_{2})+\Delta f_{0}(t_{2})$$

Frequency sensitivity in Equations (3) and (5) is set with the value *k =* 1 [14], achieving at the same time simultaneous dependence linearization *Δf*_0_ *(L*_x_ + *ΔL*_x_*)* [29,30,54]. At every switch between *Syn* signals, the frequency *f*_out_ is measured synchronously by the counter HM 8122 (Figure 1) and its value is transferred to the LabVIEW (LW) software calculating the difference between the two frequencies. The switching between *Syn* signals also highly reduces the auxiliary frequency *f*_r_ temperature instability *Δf*_r_ (*T*). This gives the frequency difference in Equation (6) representing the temperature-compensated value of the output frequency *f*_out_ depending almost uniquely on the difference between *ΔL*_x_ and *ΔL*_ref_ change.

(6) $$\begin{array}{ll} \Delta f_{out}(L_{x}) & =[f(Syn,L_{x})-(f_{r1}(T_{1})+\Delta f_{r1}(T_{1}))]\\ & -[f(\bar{Syn},L_{ref})-(f_{r2}(T_{2})+\Delta f_{r2}(T_{2}))] \end{array}$$

This means that it is virtually independent of the quartz crystal temperature characteristics *Δf*_0_ (*T*). The quartz ageing *Δf*_0_ (*t*) is practically compensated and can be ignored as the measurements are short and consecutive (a few ms). Frequency reference changes *Δf*_r_ (*T*) are also considerably reduced in Equations (6) and (7):

(7) $$\begin{array}{ll} \Delta f_{out}(L_{x}) & =\left[\frac{C}{2(\frac{1}{k}C_{0\_eff}-\frac{1}{{\omega_{0}}^{2}\cdot k\cdot L_{x}})}/2\pi \cdot \sqrt{L\cdot C}\right]-\Delta f_{0}(T_{1})-\Delta f_{r1}(T_{1})\\ & +\Delta f(cou_{err_{1}})-\left[\frac{\frac{C}{2(\frac{1}{k}C_{0\_eff}-\frac{1}{{\omega_{0}}^{2}\cdot k\cdot L_{ref}})}}{2\pi \cdot \sqrt{L\cdot C}}\right]+\Delta f_{0}(T_{2})+\Delta f_{r2}(T_{2})\\ & -\Delta f(cou\_err_{2}) \end{array}$$

### 2.4. Non-Ideality of the Temperature Compensation

As a result of the switching mode, *L*_x_ and *L*_ref_ are “alternatively” connected in series to the crystal *Q*. The frequencies *f*_01_ and *f*_02_ given by Equations (3) and (5) have different times *t*_1_ and *t*_2_ (one after the other) depending on the period of the control signal *Syn.* This means that the subtraction in Equation (7) is not performed exactly point-to-point in time. It should be mentioned that the approach has some limitations in terms of the switching times and the time-speed of the events, *i.e.*, temperature changes, whose effects can be cancelled. If these changes are sufficiently steep, temperature-related terms may not be cancelled *(Δf*_0_(*T*_1_) in Equation (3) and *Δf*_0_(*T*_2_) in Equation (5) are not equal, so they are not fully counterbalanced in Equation (7)). As a result, the lineal first order approximation of Equations (1) and (2) is no longer valid, which means that the influence of other terms is also non-negligible.

The response times of the converter, HM 8122 programmable counter and LW software determine the maximum variation temperature/time (*ΔT/Δt*) limit for which the compensation is still achieved. System (converter) response *f*_0_ *vs.* inductance variation is determined by the JFET transistor switching time (the values for the ON and OFF mode are 4 ns and 20 ns, respectively), the rise time for the NAND and NOR gates (22 ns), and the low-pass filter (LP) filter time constant which is 4.5 μs (determined by the filter *RC* components). If we take into account the response time of the two switches for one temperature compensated inductance measurement, the converter response time is ≥10 μs. The counter frequency measurement time depends on the HM 8122 software functions and the measurement mode of the LW software, as well as the speed of the instrumentation GPIB controller. To generate signal *Syn* and perform synchronous measurement an additional electronic circuit (Figure 1) was produced where the *Syn* signal is actually the *Q*_A_ output signal of the four bit binary counter. Its *CP* (clock) signal can be in the range *f* = 1–50 Hz (the speed of the measurement can be varied) and is simultaneously used as external signal triggering the HM 8122 counter (arming mode whereby the start of the measurement is delayed for 50 ns). The counter synchronously measures sequence frequency *f*_out_ (the time of one measurement is determined by the counter gate time, which cannot be less than 1 ms). For every two frequencies measured by the counter, LW software calculates the frequency difference (Equation (7)). Similarly, the frequencies *f*_01_ and *f*_02_ are sequentially measured on two HM 8122 counter channels and LW software calculates the frequency difference between Equations (3) and (5). Due to LW software communication with the HM 8122 counter and the time needed for the measurement of the two frequencies by the counter, the minimum response time is not less than 2 ms. The maximum variation temperature/time *(ΔT/Δt*) limit for which the compensation is still achieved is determined by the dynamic frequency measurement error value during the time of one *Syn* signal period, *i.e.*, within 2 ms (two sequential measurements).

### 2.5. Experimental Setup

**Figure 2.**
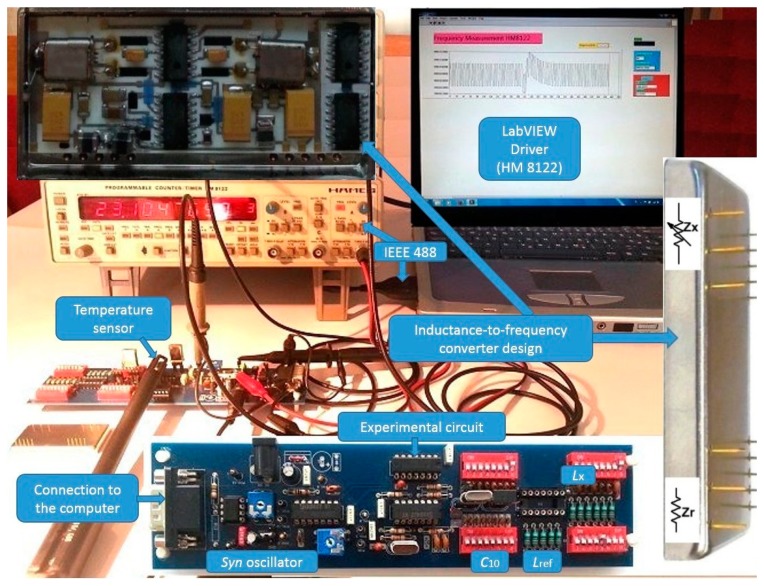
Experimental circuit and final inductance-to-frequency converter design with connection pins for industrial use.

For this experiment, first the experimental circuit (Figure 2 below) was produced. Experimental circuit is divided into two parts, where the right part is a switching section for *L*_x_, *L*_ref_ and *C*_10_ settings by dip switches (*C*_10_–*C*_12_) (Figure 1). This design was used to achieve as stable parasitic capacitances and inductances in the circuits as possible. The left part is intended for the oscillator switch time settings and an appropriate switching logic. Temperature measurements were performed by NI USB-TC01 thermocouple module (with 20 bit ADC resolution (National Instruments, Austin, TX, USA)) located near the crystal in the middle of the experimental circuit to detect temperature changes. This position is particularly important for the measurement of the dynamical stability of switching mode inductance-to-frequency converter during the temperature shocks produced by a hairdryer or uncontrolled temperature changing to which the experimental circuit is exposed. Figure 2 (left side of the experimental circuit) also shows additional oscillator, the trimmer for the switch time settings, and the connection part to the computer. Every single positive front of clock frequency (CP) (Figure 1) triggers the frequency measurement on the counter. In this way, the frequency counter works synchronously with the *Syn* signal. This frequency then serves as a clock for the binary counter. Its outputs (*Q*_A_ to *Q*_D_), which have a different time duration, serve as experimental *Syn* signal within which the counter measures the frequency *f*_out_ one or more times at the *Syn* logical conditions 1 or 0. In addition, the LW software driver also makes possible a statistical evaluation of a number of performed consecutive measurements within one *Syn* condition.

### 2.6. Inductance-to-Frequency Converter Design

Inductance-to-frequency converter was also experimentally produced in the Surface-Mount Device (SMD) technology on Al_2_O_3_ ceramics (Figure 2 (top left side and bottom right side)). At the front side of the housing, the converter has the pins for *L*_x_, *L*_ref_ (*Z*_x_, *Z*_ref_) and at the back side of the housing, it has pins for supply voltage 5V, *Sy*n signal and output frequency *f*_out_. Capacitances *L*_x_ and *L*_ref_ can be directly connected to the pins as shown in the final design of the converter. For specific industrial purposes, inductance *L*_ref_ can also be placed inside the housing. The main advantage of such a construction is that it allows the connection of the inductance sensitive elements to these pins without any additional wires with additional parasitic impedances. Connections made in this way introduce minimal parasitic inductances and parasitic capacitances, and even these are—when using switching method—reduced to the minimum.

## 3. Frequency Stability of the Converter

The factors affecting frequency stability of the converter such as wide operating temperature range, the use of various types of crystals and drive level should also be considered because a stable oscillator circuit is of vital importance. Stability of the electronic circuit depends upon the quartz crystal temperature stability and upon the circuit type and element quality (elements of the same value must be of the same quality) [12].

When using AT-cut crystals in oscillators, a frequency change in the oscillation (up to 1 Hz) of the crystal can be detected in the range between 10 and 40 °C [12,29]. Generally, different temperature frequency curves are represented as cubical parabola with temperature inflection point at 25 °C, depending on the crystal cut angle and the mechanical construction. The proposed method (Figure 1) allows the AT-cut crystal temperature characteristics compensation (under 0.1 Hz) in the above temperature range through the switching circuit and greatly reduces its influence to a minimum [44,48].

Oscillator frequency variation as a function of time is normally considered in short-term temperature stability (second-to-second) and long-term stability (ageing) over years. The short-term stability of a quartz crystal depends on the actual oscillator design and is totally controlled by the quartz crystal at low drive levels (<20 μW) [12,55]. Long-term stability is naturally greater during the first part of the crystal unit life. The ageing rates of the best cold weld crystals are less than ±1 ppm/year (10–40 °C) [13,44]. The ageing of other electronic circuit elements is compensated in the same way. If the circuit supply voltage (5 V) (Figure 1) is changed for ±1%, both frequencies *f*_01_ and *f*_02_ are changed for ±0.01 Hz, compensating the influence of the voltage change.

The reference frequency *f*_r_ is oven-controlled oscillator OCXO18T5S (Euroquartz, Somerset, UK) (4 MHz) with frequency stability of ±0.01 ppm in the temperature range of 0°– +60 °C following the warm-up time of 1 min [44]. Bear in mind that for industrial use a less stable oscillator can be used. Through *Syn* signals, the output frequency *f*_out_ also reduces the influence of the reference frequency change.

## 4. Frequency Measurement Error

Frequency changes *Δf*_c1_ (cou_err_1_) and *Δf*_c2_ (cou_err_2_) in Equations (1) and (7) are undefined, and presented here as a part of these equations. They in fact include both the counter measurement error and oscillator noise because it is very difficult to differentiate between the two. The frequency measurement errors can differ at every single measurement [53]. Different oscillator noises (Phase Modulated (PM), jitter and thermal Johnson) are all included in *Δf*_c._ The frequency is measured by the programmable counter HM8122 (with the accuracy of ±5 × 10^−9^ (through the entire working temperature range of 10–40 °C)).

Switching mode method first and foremost compensates (considerably reduces) quartz crystal temperature influence. This influence is significantly greater than those of the noise and counter accuracy.

## 5. Experimental Results

For this experiment, a prototype electronic circuit was produced guaranteeing physically stable conditions at inductances *L*_x_, *L*_ref_ and capacitance *C*_10_–*C*_12_ (Figure 1). Stable parasite capacitances and inductances assure repeatability of the experimental results [56–58].

### 5.1. Inductance-Frequency Characteristics of the Oscillator

**Figure 3.**
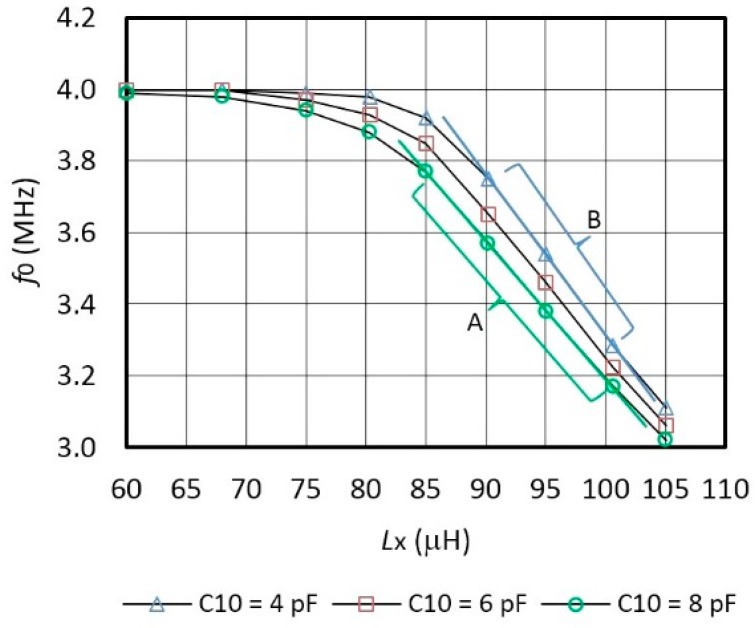
Inductive frequency characteristics *f*_0_ (for different values of capacitance *C*_10_ in parallel to the crystal at T = 25 °C).

Figure 3 shows oscillator's frequency characteristics *f*_0_ with regard to the change of the inductance *L*_x_ and a comparison of the characteristics for various sensitivity values *C*_10_ = (4 pF, 6 pF, 8 pF) connected in parallel to the quartz crystal. The capacitance *C*_10_ = 8 pF records the sensitivity of 39 kHz/μH (Figure 3 (linear area A)) in the range *L*_x_ = 85–100 μH. When *C*_10_ = 4 pF, the sensitivity is 43 kHz/μH (Figure 3 (linear area B)) in the range *L*_x_ = 90–100 μH. The settings of *L*_x_ are in steps of 60 μH, 68 μH, 75 μH, 80.3 μH, 85 μH, 90.2 μH, 95 μH, 105 μH, and 100.6 μH.

However, for this particular experiment, the capacitors *C*_10_ and inductances *L*_x_ with tolerance of 0.1% were specially selected [56–58] by the measurement with HP 4194A impedance/gain phase analyzer.

### 5.2. Non-Compensated and Compensated Frequency/Temperature Variation of the Oscillator

**Figure 4.**
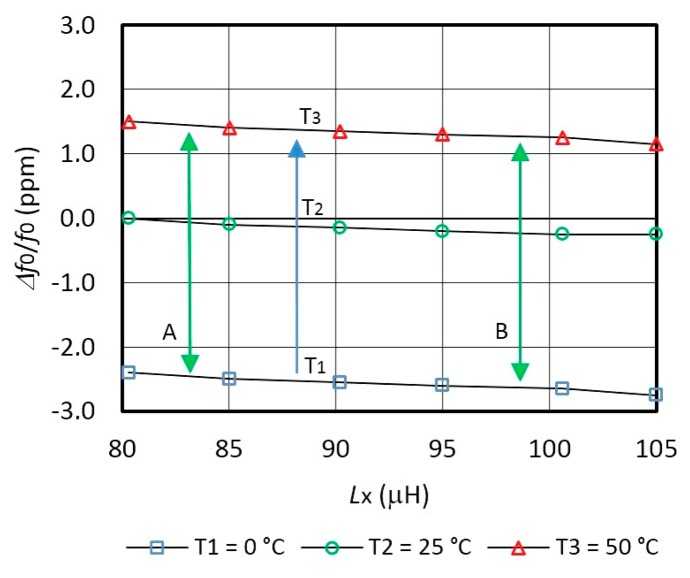
Non-compensated oscillator frequency *Δf*_0_/*f*_0_ characteristics variation depending on the temperatures *T*_1_, *T*_2_ and *T*_3_ and on *L*_x_ (*C*_10_ = 8 pF).

Figure 4 shows non-compensated *Δf*_0_/*f*_0_ frequency variations for different inductances *L*_x_ for three temperatures *T*_1_ = 0° C, *T*_2_ = 25 °C, and *T*_3_ = 50 ° C. When the inductance *L*_x_ = 0 μH, a typical frequency variation for the AT-cut crystal (at *T*_1_, *T*_2_ and *T*_3_) is −2.5 ppm at *T*_1_ = 0 °C and 1.5 ppm at *T*_3_ = 50 °C depending on the reference temperature point *T*_2_ = 25 °C [8]. By increasing inductance value *L*_x_, frequency/temperature variation is changed due to non-ideal inductances. Temperature control was performed by programmable climate chamber Weiss SB1 160.

**Figure 5.**
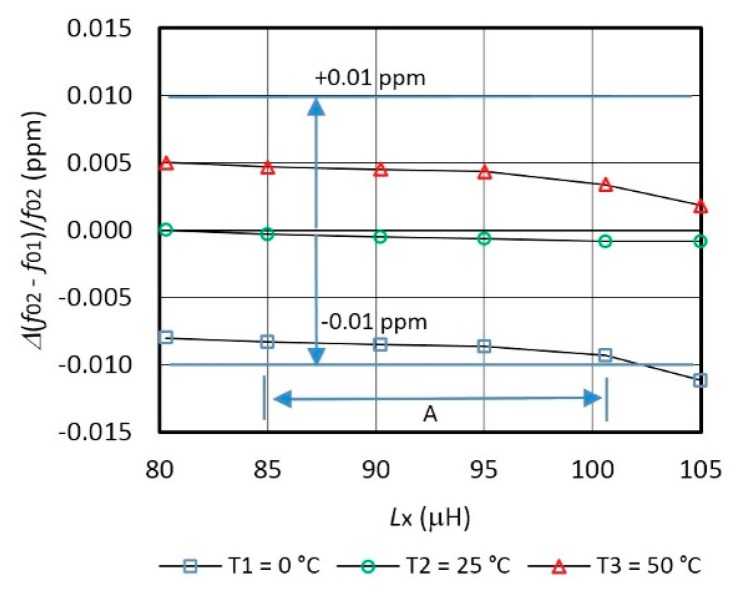
Switching mode compensated oscillator` s frequency *Δ*(*f*_01_ − *f*_02_)/*f*_02_ characteristics variation depending on the temperatures *T*_1_, *T*_2_ and *T*_3_ and on *L*_x_ (*C*_10_ = 8 pF, *L*_ref_ = 85 μH, *f*_Syn_ = 1 Hz).

Figure 5 shows switching mode frequency variation *Δ*(*f*_01_ − *f*_02_)/*f*_02_ in the range ±0.01 ppm after the temperature compensation. The comparison of the Figures 4 and 5 points to the suitability of the proposed approach.

**Figure 6.**
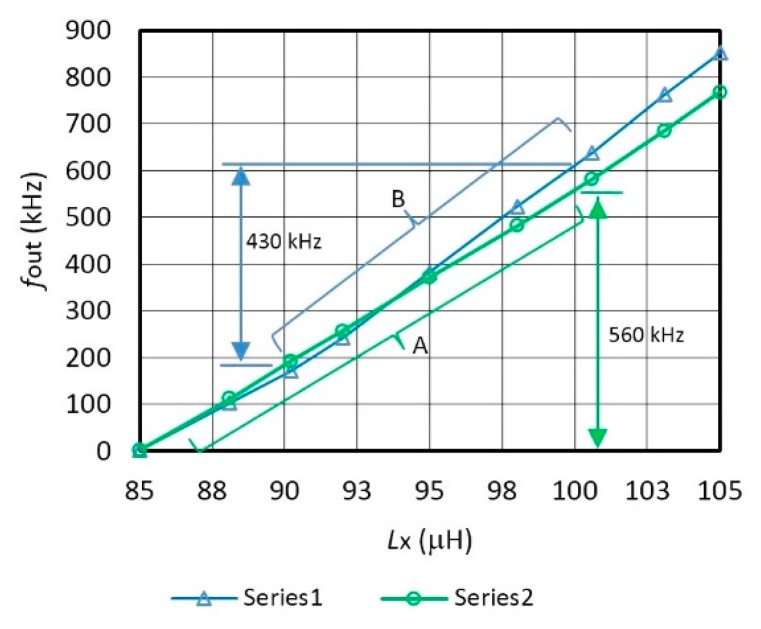
Compensated frequency characteristics for two different values of *C*_10_ at *L*_ref_ = 85 μH.

Figure 6 shows switching mode compensated output (*f*_out_) inductance-frequency characteristics of the converter with regard to the change of the capacitance *L*_x_ and a comparison of the characteristics for two capacitances *C*_10_. The capacitance *C*_10_ = 8 pF records the highest linearity of 0.1% of the inductance-frequency characteristics in the range 85–100 μH, where the frequency change is 2–560 kHz (Figure 6A), while when *C*_10_ = 4 pF it has this linearity in the range 90–100 μH and the frequency change of 430 kHz (Figure 6B). The setting of *L*_x_ is in steps of 85 μH, 88.1 μH, 90.2 μH, 92 μH, 95 μH, 98 μH, 100.6 μH, 103.1 μH, and 105 μH which have tolerance of 0.1%.

### 5.3. Switching Mode Temperature Dynamic Stability

**Figure 7.**
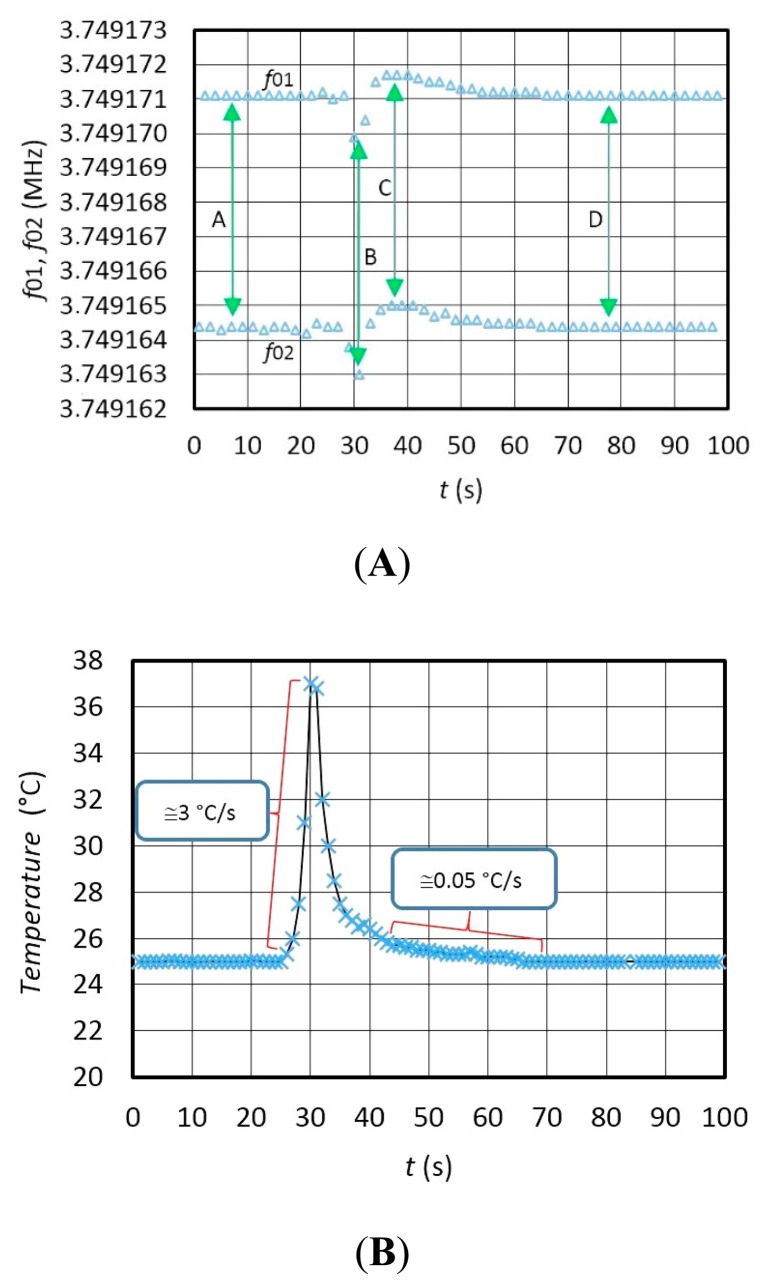
(**A**) Extended temperature dynamic frequency instability for *f*_01_ and *f*_02_; (**B**) temperature shock (25 °C–37 °C). *f*_Syn_ = 1 Hz.

Figure 7 experimentally shows the switching mode extended dynamic instability—the frequencies change of *f*_01_ and *f*_02_ (Figure 7a) if the converter is influenced by a temperature changing from 25 °C–37 °C (temperature shock produced by a hairdryer) (Figure 7b). Figure 7a demonstrates that the temperature influence on the *f*_01_ and frequency *f*_02_ (in the time span between 0 and 100 s) changes the frequency difference *f*_01_ and *f*_02_ in the same size class (A, B, C, D). During the temperature shock, the speed of temperature variation is ≅ 3 °C/s in the time span of 25–30 s, and ≅0.05 °C/s in the time span of 45–70 s at slower cooling (Figure 7b). The temperature in the immediate vicinity of the crystal was measured at every *Syn* period with the NI USB-TC01 thermocouple measurement device (National Instruments, Austin, TX, USA) synchronously with the LW software. The frequency shift between *f*_01_ and *f*_02_ depends on the difference between *L*_x_ and *L*_ref_ (*L*_x_ = 85.001 μH and *L*_ref_ = 85 μH).

### 5.4. Converter Output Temperature/Frequency Dynamic Error

**Figure 8.**
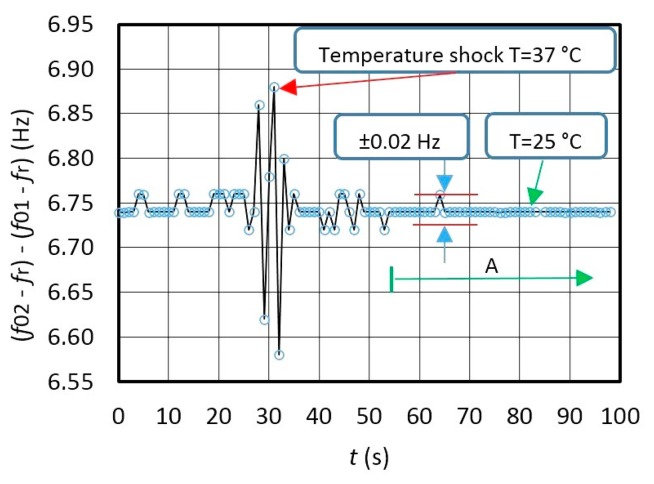
Output frequency dynamic variation of *f*_out_ during the change of temperature from 25 °C to 37 °C and back to 25 °C (measurement time: 0–100 s, *f*_out_ = *f*_02_ − *f*_01_, *L*_x_ = 85.001 μH, *L*_ref_ = 85 μH).

Figure 8 illustrates frequency variation for *f*_out_ = (*f*_02_ − *f*_r_) − (*f*_01_ − *f*_r_) at frequency difference ≅ 6.74 Hz between *f*_02_ and *f*_01_ (*f*_r_ = 2 kHz) during the temperature change (Figure 7) in the range 25–37 °C determined by the fixed *L*_x_ and *L*_ref_ (Figure 7). Deduction of both frequencies in relation to *Syn* signals is performed by LW software. The latter also shows high frequency dynamic stability (Figure 8) (A) in the range ±0.02 Hz), in which the environment temperature does not change so quickly anymore (Figure 7b − *ΔT/Δt* = 0.05 °C/s).

The comparison of the results in Figures 7A and 8 shows that the dynamic temperature influence on the frequency change *f*_02_–*f*_01_ (Figure 7A is approximately the same and well dynamically compensated at the output of the converter as illustrated by Figure 8.

**Figure 9.**
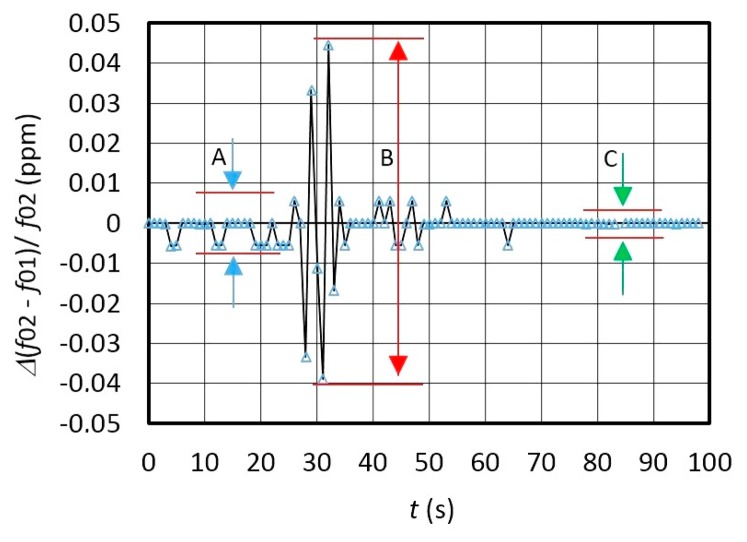
Output frequency dynamic error *Δf*_out_ (ppm) during the change of temperature from 25 °C to 37 °C and back to 25 °C (Figures 7 and 8).

Figure 9 shows dynamic error of the frequency difference change *f*_01_ and *f*_02_ in ppm measured by the counter and calculated by LW software every single second. The results show that the frequency difference variation *Δf*_out_ = *Δ*(*f*_02_ − *f*_01_)/*f*_02_ has three different values A, B and C. A in Figure 9 illustrates the change of the frequency variation at T = 25 °C ± 0.5 °C, B shows the temperature shock causing the temperature to rise from 25 °C to 37 °C and then drop back to 25 °C, and C illustrates the case without any temperature shock at T = 25 °C ± 0.2 °C. This is shown on Figure 8 (area A) where the output frequency stability is in the range ±0.01 ppm (Figure 9), which proves the applicability of this method for the inductance measurements in the pH range. In case the consecutive measurements are performed in a time shorter than 1 s, the variation B (Figure 9) is smaller (explained in greater detail in Section 2.4). However, it is worth mentioning that converter's own response time is just 10 μs enabling swifter consecutive measurements.

If the output frequency sensitivity *f*_out_ ≅ 39 kHz/μH (Figure 6) is in the temperature range between 10 and 40 °C, the supply voltage stability is 5 V ± 0.01 V, and the frequency reference *f*_r_ stability is 0.005 ppm, then dynamic frequency stability at the output *f*_out_ = ±0.002 Hz, which gives the converter resolution of ±2 pH.

## 6. Conclusions

The experimental results show that the switching method excellently reduces the influence of quartz crystal non-linear frequency-temperature characteristics, its ageing and that of oscillator circuit elements, the influence of the supply voltage on the oscillating circuit, as well as the reference frequency temperature instability. The greatest advantage of the proposed method is that it resolves the issue of high sensitivity and linearity, and reduces the temperature influence of the main oscillating element (quartz crystal) to a minimum at the same time. The experimental results shown in the article relate to a significantly wider frequency range (2–100 kHz when using LP filter, and 2–560 kHz when using direct measurement of both frequencies by frequency counter) than is usually covered by practical measurements for inductance range 80–100 μH with pH resolution.

The results clearly show that the oscillator switching method for high-precision inductance-to-frequency transducing opens up new possibilities through the compensation of the main oscillating element self-temperature and minimization of other frequency variation influences. This makes this switching method a very interesting tool for the inductance-to-frequency converter especially because of the pH resolution which is highly promising in various fields of physics, chemistry, magnetic material properties measurement, mechatronics, and biosensor technology and in specific high-quality production industries.
